# Irreversibility transition of colloidal polycrystals under cyclic deformation

**DOI:** 10.1038/srep45550

**Published:** 2017-03-30

**Authors:** Pritam Kumar Jana, Mikko J. Alava, Stefano Zapperi

**Affiliations:** 1 COMP Centre of Excellence, Department of Applied Physics, Aalto University, P.O.Box 11100, FI-00076 Aalto, Espoo, Finland; 2 Center for Complexity and Biosystems, Department of Physics, University of Milano, via Celoria 16, 20133 Milano, Italy; 3 ISI Foundation, Via Alassio 11/C, 10126, Torino, Italy; 4 CNR-ICMATE, Via R. Cozzi 53, 20125, Milano, Italy

## Abstract

Cyclically loaded disordered particle systems, such as granular packings and amorphous media, display a non-equilibrium phase transition towards irreversibility. Here, we investigate numerically the cyclic deformation of a colloidal polycrystal with impurities and reveal a transition to irreversible behavior driven by the displacement of dislocations. At the phase transition we observe enhanced particle diffusion, system size effects and broadly distributed strain bursts. In addition to provide an analogy between the deformation of amorphous and polycrystalline materials, our results allow to reinterpret Zener pinning of grain boundaries as a way to prevent the onset of irreversible crystal ordering.

Periodically driven disordered granular media and colloidal suspensions display an intriguing transition from reversible to irreversible deformation as the amplitude of the loading overcomes a threshold value^1^,^2^,^3^,^4^,^5^,^6^,^7^,^8^. A very similar behavior is also observed in simulations of amorphous solids under cyclic shear^9^,^10^,^11^, suggesting that it might be a general feature of slowly relaxing disordered systems. These results are interesting not only because they help to clarify how irreversible behavior arise from reversible microscopic dynamics^1^ but also because they shed light on the nature of yielding in amorphous media^12^. In the light of these results, we can interpret the onset of plasticity as a phase transition from a reversible elastic dynamics to an irreversible plastic deformation. The relation between yielding in crystals, where irreversibility is associated to the motion of defects, and glasses is still a subject of current investigation.

Colloidal suspensions are becoming a widely used tool to study experimentally the properties of matter since the change of scale from the atomistic to the colloidal ones allows to directly observe phenomena that would otherwise not be within experimental reach. Depending on the experimental conditions colloidal particles can be assembled into desired states, from perfectly ordered crystals^13^ to disordered amorphous glasses^14^. In between these two extremes lie colloidal polycrystals, where ordered crystalline regions are separated by extended grain boundaries formed by dislocation arrays^15^,^16^,^17^,^18^. The application of a cyclic shear to colloidal polycrystals allows to follow at the same time the dynamics of individual particles (“atoms”) and the large-scale response of the polycrystalline texture^15^,^16^,^17^,^18^.

Here, we investigate the response of colloidal polycrystals to cyclic loading by molecular dynamics simulations and explore the coupling between dislocations, grain boundaries and the dynamics of individual colloidal particles. The system we consider is similar to the experimental setup studied in ref. 18, where a colloidal crystal is doped with larger colloidal particles whose concentration is used to control the grain size. Our simulations show that for large enough strain amplitudes grain boundaries eventually disappear and the system orders, while at small amplitude the system settles after an initial transient to a state very close to the original metastable configuration and reversibility is maintained. Thus, there is an effective phase transition controlled by the strain amplitude between asymptotic steady-states. The dynamics of this transition is found to be of a transient character, and leads to the presence of crackling noise in the system response. We also show that higher impurity concentration leads to a larger yield strain. The depinning of grain boundaries or cross-over to irreversibility is a non-equilibrium version of the Zener pinning in grain boundary coarsening in metals in the presence of impurities^19^,^20^.

## Results and Discussion

**Figure 1:**
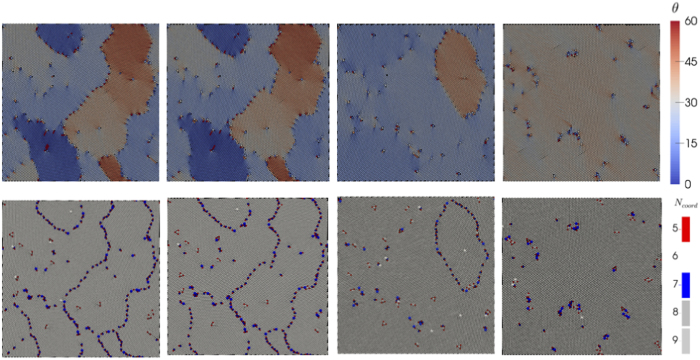
Grain orientations and boundaries before and after the applied strain. Top and bottom panels correspond to the configurations with local orientation *θ* and nearest neighbours *N*_*coord*_, respectively. An initial configuration is depicted in the first column. The other configurations are (after *N* = 600 cycles) for *γ*_0_ = 0.031, *γ*_0_ = 0.056, and for *γ*_0_ = 0.069, respectively.

In 2D triangular colloidal crystals, five coordinated particle, known as positively charged disclinations (+1), and seven coordinated particles, corresponding to negatively charged disclinations (−1), exist as defects. The likelihood of an individual disclination occurring is low. However, the combination of five and seven coordinated particles with a small additional energy creates edge dislocations. A regular array of dislocations then creates a grain boundary separating differently oriented crystals, as shown in Fig. 1. The top panel in Fig. 1 corresponds to the local crystal orientation, *θ*, and the bottom panel represents the nearest neighbour characteristics, *N*_*coord*_, of each particle. A careful study shows that arrays of dislocations are mainly arranged along the grain boundaries with high misorientations, which is consistent with Frank condition, *n* ∝ sin *dθ*, where *n* is the line density along the grain boundary with the corresponding misorientaion *dθ*^21^,^22^.

**Figure 2:**
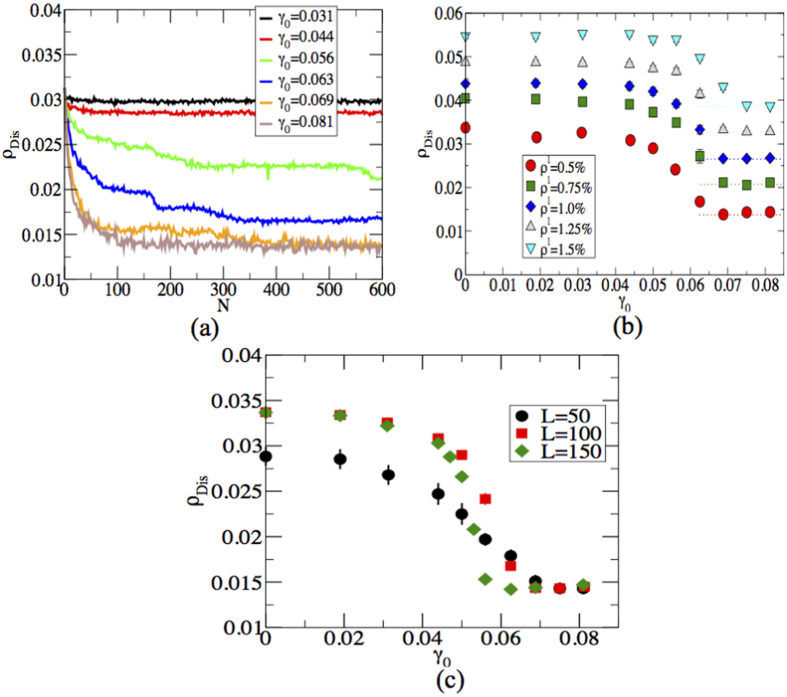
Discliantion density. (**a**) Shows the time evaluation of disclination density *ρ*_*Dis*_ and (**b**) displays *ρ*_*Dis*_ as a function of *γ*_0_ for different values impurity density *ρ*^*l*^. Dotted lines exhibit the steady state values of disclination density. (**c**) *ρ*_*Dis*_ as a function of strain amplitude for different values of system sizes keeping *ρ*^*l*^ fixed at 0.5%.

Once the polycrystalline samples are prepared, they undergo cyclic deformation with different values of strain amplitude *γ*_0_. For low values of *γ*_0_, the grain boundary network recovers its initial configuration after each cycle (see Fig. 1 second column and Video S1 in Supplementary information), whereas for higher values of *γ*_0_ all dislocations annihilate from the initial polycrystalline samples (see Fig. 1 last column and Video S3 in Supplementary information). For intermediate values of *γ*_0_, however, a memory of the initial grain boundary structure can still be observed after cycling (see Fig. 1 third column and Video S2 in Supplementary information). To quantify the response to cyclic shear, we measure the time evaluation of the disclination density *ρ*_*Dis*_, defined as the number density of particles having either 5 (+1 disclination) or 7 (−1 disclination) neighbours, for different values of *γ*_0_ as shown in Fig. 2(a). For low values of *γ*_0_, *ρ*_*Dis*_ remains unchanged which is consistent with the previous discussions. However, when *γ*_0_ > 0.044, *ρ*_*dis*_ rapidly decreases and reaches a steady state value. When the impurity concentration increases, a large value of strain amplitude is required to reach the asymptotic disclination density, which is shown in Fig. 2(b). It could be understood as follows: from Fig. 2(b), disclination density increases by 66.7% when impurity density is increased by 200% that explains the increase of impurity density along the grain boundaries. Segregation of impurities in the grain boundaries leads to lowering of total free energy^23^,^24^ and therefore the increase of yield stress is observed. The asymptotic disclination density *ρ*_*Dis*_ corresponds to the disclinations created by the presence of larger particles and can be estimated as *cρ*^*l*^, where *c* ≈ 2.5 and *ρ*^*l*^ is the density of the larger particles. We notice that the final disclination density after 600 cycles depends on the sample size and the transition becomes sharper when *L* is larger. See Fig. 2(c).

**Figure 3:**
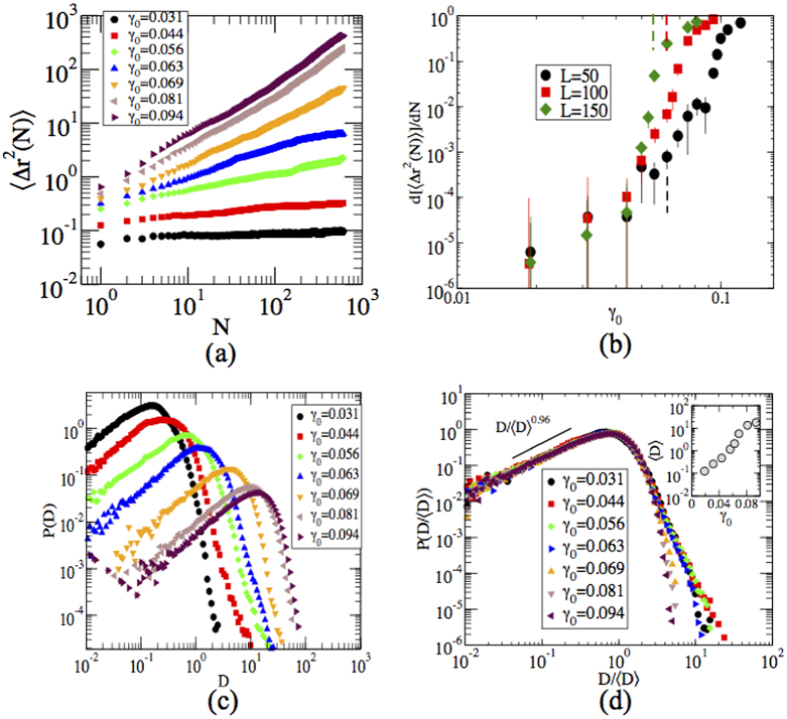
Dynamical heterogeneity. (**a**) Mean square displacement, 〈Δ*r*^2^(*N*)〉, as a function of number of cycles, *N*, for different values of strain amplitude *γ*_0_. (**b**) Diffusivity, 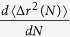, are plotted as a function of *γ*_0_ for three different system sizes. Dotted lines correspond to 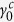 values. (**c**) Probability distribution of the particle displacements (*D*) measured at the end of 600 cycles. (**d**) Shows a collapse of the plots shown in (**c**), into a single master curve. The inset shows the scaling of the 〈*D*〉 with *γ*_0_.

In order to understand the behavior of the dynamics at the particle level and the role of reversibility and irreversibility, we calculate the mean square displacements (MSDs), Δ*r*^2^, with respect to the initial configurations averaged over 100 samples. Figure 3(a) shows that for small values of *γ*_0_, Δ*r*^2^ saturates after initial transient, whereas for higher values of *γ*_0_, the particle displacements become diffusive. That is, for large enough values of *γ*_0_, MSD increases in a linear way, implying irreversible single particle behavior. When the system comes to the related steady state, one can extract the diffusivity, 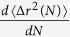, which is plotted in Fig. 3(b) for different system sizes. When *γ*_0_  ⩽0.044, the system remains in a quiescent state, however for larger values of *γ*_0_, diffusivity increases sharply. In short, both the quantities indicate that the colloidal polycrystals respond differently depending on the amplitude of *γ*_0_.

In order to measure the dynamical heterogeneity in the system, we compute the probability distribution function (PDF) of the particle displacement magnitude (D) after *N* = 600 cycles with respect to the initial configurations for different values of *γ*_0_ (see Fig. 3(c)). Histograms for different values of *γ*_0_ are collapsed by rescaling *D* with average displacement 〈*D*〉. The first part of the collapsed data in Fig. 3(d) scales as 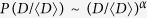 and α = 0.96, i. e. the exponent is very close to unity. The tail part exhibits an exponential decay when *γ*_0_ > 0.0625 and for smaller values decays very rapidly. It is interesting to note that both for small and big *γ*_0_-amplitudes the histograms are in any case quite similar and that Fig. 3(c) connects the reversibility-irreversibility change to the largest typical values of *D* found: when these start to be of the order of unity, reversibility is lost. This is seen also by looking at the Inset of Fig. 3(d). The single-particle behavior averaged and measured by 〈*D*〉 shows no direct signs of collective behavior, but as the displacement magnitude grows - and reversibility is destroyed - collective effects and restructuring starts.

In order to understand the plastic deformation at the particle level, we compute the local shear strain, 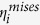, for each atom at the end of each cycle^25^. To this end, we consider the initial configurations as references and find the local transformation matrix ***J***_*i*_ that best maps 
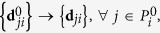
 where **d**’s are vector separations (row vectors) between atom *j* and *i* (superscript 0 means the reference configuration). Here, *j* is one of atom *i*’s nearest neighbours, and 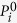 is the total number of nearest neighbours of atom *i*, at the reference configurations. **J**_*i*_ is determined by minimising 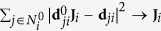. For each **J**_*i*_, the local Lagrangian strain matrix is computed as 
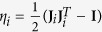
. Then local shear invariant is calculated for each atom *i* as follows


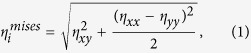


and average is taken over all atoms present in the system.

**Figure 4:**
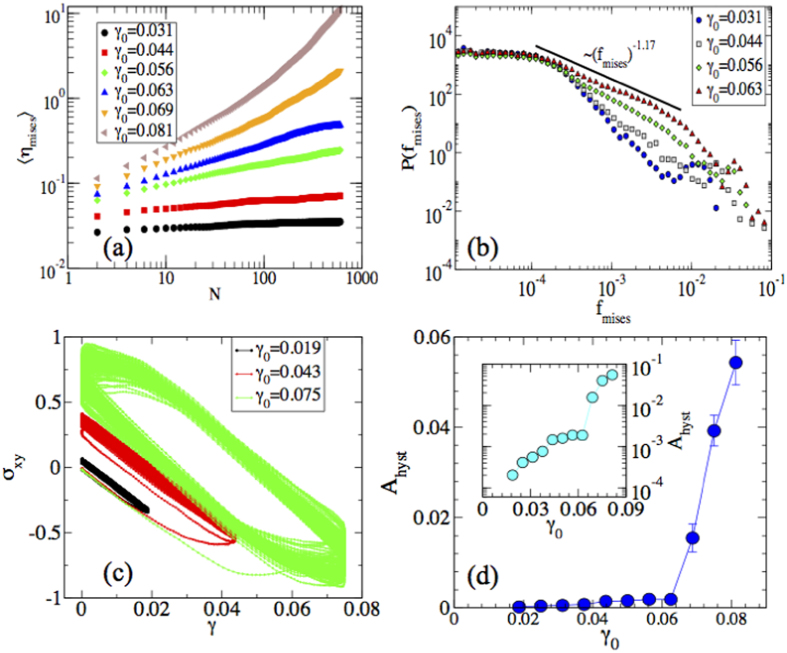
Plastic deformation. (**a**) Average shear strain 〈*η*_*mises*_〉 is shown as a function of *N* for different values of strain amplitude *γ*_0_. (**b**) Probability distribution of *f*_*mises*_ for different values of strain amplitude *γ*_0_. (**c**) Shear stress *σ*_*xy*_ is shown as a function of applied strain *γ*. (**d**) Area of the hysteresis loop *A*_*hyst*_ obtained from (**c**) is plotted against strain amplitude. Inset shows the log-lin plot for the clarity.

**Figure 5:**
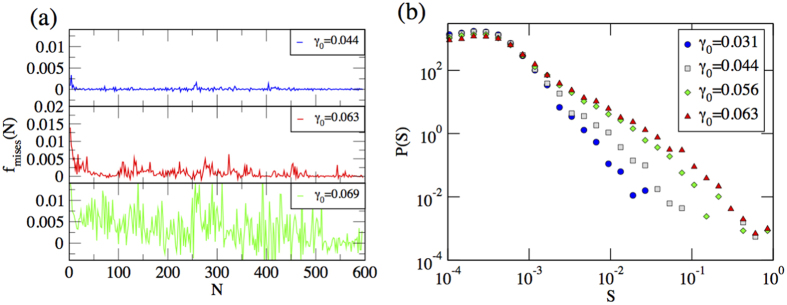
Avalanache signal and sizes. Left panel: avalanche signal for three values of *γ*_0_. Right panel: avalanche size (*S*) distribution for different values of *γ*_0_.

First, we have plotted 〈*η*_*mises*_〉 versus *N* as shown in Fig. 4(a) which has qualitatively similar behavior as 2(a). The strain shows large, transient fluctuations close to the transition, as plotted in Fig. 5(a) where we plot the strain rate (*d*〈*η*_*mises*_〉)/(*dN*), denoted as *f*_*mises*_, as a function of number of cycles for different values of *γ*_0_. Note how the strain signal evolves with *N* for the two larger amplitudes. This crackling noise is the signature of the non-equilibrium transition of grain boundary depinning which can be clearly seen in Videos S2 and S3 in Supplementary Information. We then compute the strain rate distribution and find that it exhibits a scaling as 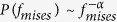 with *α* = 1.17 for *γ*_0_ = 0.063 (see Fig. 4(b)). However, with decreasing the strain amplitude towards *γ*_0_ = 0.031, the exponent *α* increases. This is because for lower values of *γ*_0_, large events are less likely as shown in *f*_*mises*_(*N*) vs *N* plot. To quantify the energy dissipation during the plastic events, hysteresis loop area is computed from the shear stress against applied strain as shown in Fig. 4(c) and plotted as a function of strain amplitude (see Fig. 4(d)) where transition is attributed by observed energy dissipation. In a similar study Laurson *et al*.^26^ has characterized two phases corresponding to the jammed and the moving state by computing hysteresis loop area when the crystalline solids with dislocations are stressed cyclically. It has been explained in the light of collective dislocation dynamics.

To quantify the statistics of crackling noise in colloidal crystal, we compute the distribution of avalanche sizes which is defined as the sequence of values of *f*_*mises*_(*N*) exceeding some threshold value 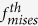 to subtract an uncorrelated back ground. If an avalanche starts at *N* = 0 and ends at *N* = *n*, the size of an avalanche of duration *n* is defined as


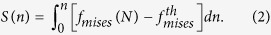


**Figure 6:**
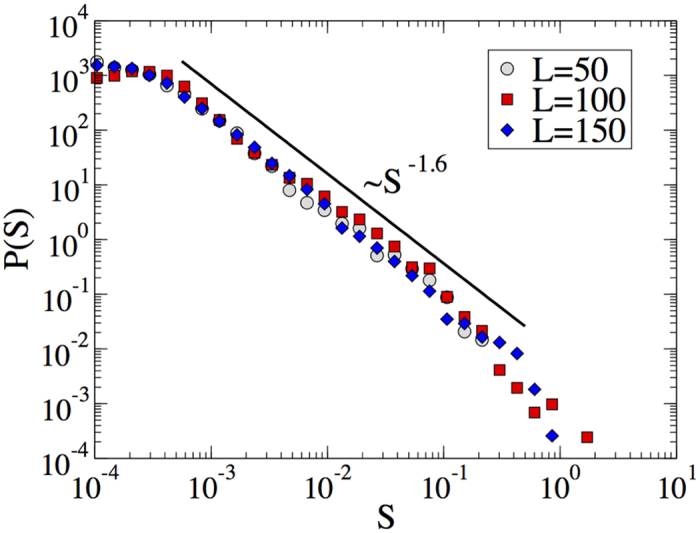
Avalanche size for different system sizes. Avalanche size distribution for different system sizes for strain amplitude *γ*_0_ = 0.063 in case of system sizes *L* = 50 and *L* = 100 and for *γ*_0_ = 0.05 when the system size is *L* = 150.

**Figure 7:**
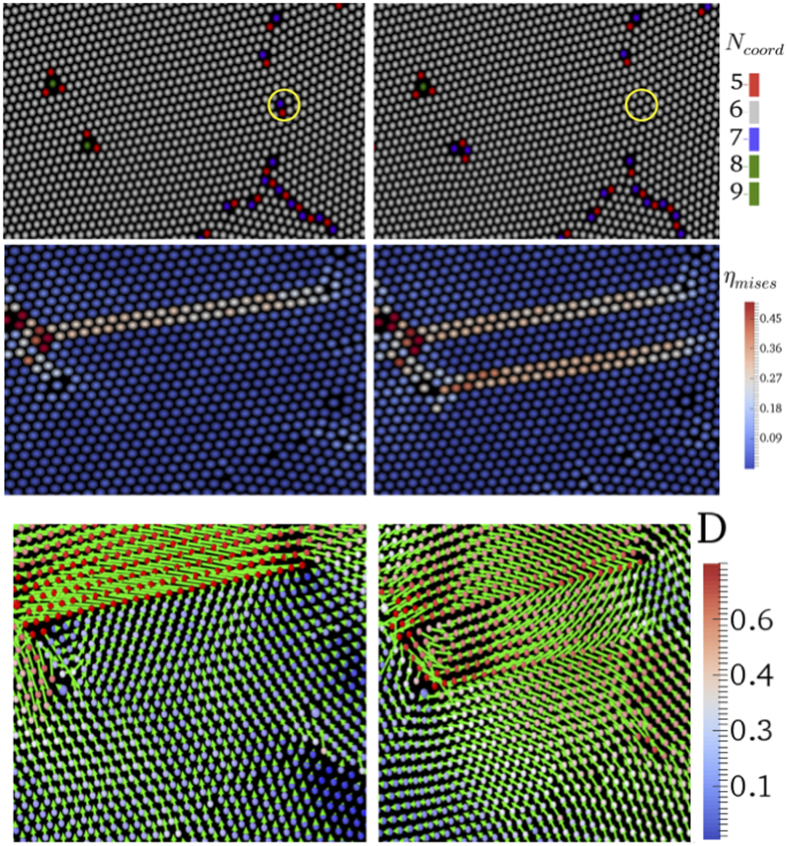
Dislocation movement. Top panel: dislocation indicated by yellow circle slides through the crystal until it meets another defect. Middle panel: A signature of sliding motion is seen in *η*_*mises*_ calculations. Below panel: Displacement map corresponding to the sliding motion. Arrow indicates direction and magnitude of the displacement. For more clearance, a color legend of the magnitude has also been presented.

Distributions of avalanche sizes are reported in Fig. 5(b). As expected in general for crackling noise, the distribution crucially depends on the strain amplitude. For higher values of *γ*_0_, the avalanche distribution decays as a power law, *P(S*) ~ *S*^−*τ*^, where *τ* = 1.57 and the exponent *τ* increases as we move towards the lower values of strain amplitudes. The clearest power law is observed at the critical point *γ*_0_ = 0.063 where the cutoff increases with the system size *L* as shown in Fig. 6. These distributions result from an integration of the bursty dynamics (Fig. 5) which is of transient character. Eventually after an amplitude-dependent number of cycles the noise develops a stationary character. In passing notes, we also observe sliding of dislocations and rotation of grains which induce the plastic deformations in polycrystalline materials as described in ref. 27. See Fig. 7.

## Summary

In conclusion, we have simulated a colloidal polycrystal under cyclic shear and observed a transition from reversible deformation in which the grain structure is preserved after each cycle and an irreversible phase leading to crystal ordering and the disappearance of grain boundaries and dislocations. The transition displays all the typical signs of other non-equilibrium phase transitions such as finite size effects and power law distributed crackling noise. Our results indicate that colloidal polycrystals under oscillatory shear behave similarly as disordered particle assemblies and colloidal glasses, but the relevant degrees of freedom are here the topological defects instead of the particles themselves. The behavior we uncover has direct application to Zener pinning where inclusions are used to stop grain growth in a polycrystal^19^. The critical strain amplitude for irreversibility should correspond to the depinning stress for grain boundary growth^20^. Our results should be directly testable in colloidal particle experiments^15^,^16^,^17^,^18^.

## Methods

We consider a two-dimensional system with two types of particles, large (*l*) and small (*s*) interacting via a pair-wise Lennard-Jones potential


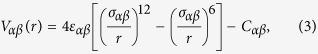


where *r* is the distance between a particle of type *α* and one of type *β(α, β* = *l, s*). The functions *V*_*αβ*_(*r*) = 0, when 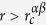 a cutoff distance, which is defined by 3.0_*σαβ*_ and the constant *C*_*αβ*_ ensures the continuity of *V*_*αβ*_ at 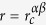. The parameters of the potential are chosen as follows: *σ* ≡ *σ*_*s*_ = 1.0*, σ*_*l*_ =* *1.4*σ* and *σ*_*ls*_ = 1.2*σ* are the particle diameters and *ε* *≡* *ε*_*ll*_ =* *1.0*, ε*_*ss*_ = 0.5*ε, ε*_*ls*_ =* *1.5*ε. r*_*c*_ =* *3.0*σ* are the energy parameters. In what follows, the lengths are normalized by *σ*. The typical numbers of the particles used in the system are *N*_*l*_ = 50 and *N*_*s*_ = 10000, with *m* = 1 for both with 100 samples for ensemble averaging over the initial conditions. The typical length of the 2D simulation box is *L* = 100. The unit of time is set 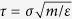. The equations of motion are solved numerically using the time-reversible measure-preserving Verlet and rRESPA integration scheme with a time step Δ*t* *=* 0.005*τ*. To control the temperature the system is connected with Nose-Hoover thermostat and Lees-Edward boundary conditions are used to apply shear.

A polycrystalline structure, as shown in the top left of Fig. 1 (color shows the orientation of the crystals), is prepared as follows: the system is heated at *T* = 2.0*ε/k*_*B*_ for the duration of 255 *τ*. Temperature is decreased to 0.01 *ε/k*_*B*_ by a span of 150*τ* and the system is equilibrated for a time 5 × 10^3^ *τ*. Then, the system undergoes cyclic deformation along the *x*-direction with the following way: the system is sheared for 50 *τ* after which a similar reverse step is applied. The cycle is started and finished by a relaxation period of duration of 5 *τ*. A full cycle thus lasts 110 *τ* and the data presented below considers up to *N* = 600 cycles. To study the system size dependence, we consider systems of size *L* = 50 and *L* = 150 in addition to *L* = 100. To obtain a same strain amplitude for different sample sizes, the duration of shear phases are changed from 50 *τ* to 35.36 *τ* and 61.24 *τ* for *L* = 50 and *L* = 150, respectively. However, the relaxation time remains same for all the system sizes. Therefore, for *L* = 50 and *L* = 150, a full cycle consists of 80.72 *τ* and 132.48 *τ*, respectively. Molecular dynamics simulations using the above mentioned prescriptions are performed with LAMMPS (Large-scale Atomic/Molecular Massively Parallel Simulator: a free, open source Molecular Dynamics Simulators)^28^.

## Additional Information

**How to cite this article:** Jana, P. K. *et al*. Irreversibility transition of colloidal polycrystals under cyclic deformation. *Sci. Rep.* **7**, 45550; doi: 10.1038/srep45550 (2017).

**Publisher's note:** Springer Nature remains neutral with regard to jurisdictional claims in published maps and institutional affiliations.

## Supplementary Material

Supplementary Video S1

Supplementary Video S2

Supplementary Video S3

Supplementary Information
