# Supplementary material for: Irreversibility transition of colloidal polycrystals under cyclic deformation
Source: Sci Rep. 2017 Mar 30;7:45550. doi: 10.1038/srep45550 (PMC5372088; doi:10.1038/srep45550)
Supplement: Supplementary Information [file srep45550-s4.pdf]

Supplementary information

Irreversibility transition of colloidal polycrystals under cyclic deformation

Pritam Kumar Jana, Mikko J. Alava, and Stefano Zapperi

Title of file: Video S1
Video title: Evaluation of grain boundary network for a low value of strain amplitude γ_0=0.031.
Video legend: Same as bottom panel in Fig. 1.

Title of file: Video S2
Video title: Evaluation of grain boundary network for an intermediate value of strain amplitude γ_0=0.056.
Video legend: Same as bottom panel in Fig. 1.

Title of file: Video S3
Video title: Evaluation of grain boundary network for a large value of strain amplitude γ_0=0.069.
Video legend: Same as bottom panel in Fig. 1.
